# Characteristics of bloodstream infection and initial antibiotic use in critically ill burn patients and their impact on patient prognosis

**DOI:** 10.1038/s41598-022-24492-z

**Published:** 2022-11-22

**Authors:** Zhang Yin, Wu Beiwen, Ma Zhenzhu, Chen Erzhen, Zhang Qin, Dou Yi

**Affiliations:** 1 Department of Burn, Ruijin Hospital, Shanghai Jiaotong University School of Medicine, Shanghai, 20025 China; 2 School of Nursing, Shanghai Jiaotong University, Shanghai, 200025 China; 3 Department of Nursing, Ruijin Hospital, Shanghai Jiaotong University School of Medicine, Shanghai, 200025 China; 4 Department of Emergency, Ruijin Hospital, Shanghai Jiaotong University School of Medicine, Shanghai, 200025 China

**Keywords:** Infection, Antimicrobials, Bacterial infection, Trauma

## Abstract

To investigate the bacterial epidemiology of blood cultures taken during the treatment of critically ill burn patients, the use of antibiotics at admission and before the observation of positive blood cultures, and their effect on prognosis. A retrospective study method was used. From January 1, 2010, to December 31, 2019, burn patients who met the inclusion criteria and were treated at the Burn Department, Ruijin Hospital, Shanghai Jiaotong University School of Medicine, were enrolled in the study. Data were collected from the patients’ electronic medical records. General patient information, including length of hospital stay, length of intensive care unit (ICU) stay, in-hospital mortality, the bacteria epidemiological characteristics of blood cultures, and the use of antibiotics within 24 h after admission and before a positive blood culture was observed, was collected. Independent sample t tests and *χ*^*2*^ tests were used to compare the effects of a positive blood culture and the use of appropriate antibiotics within 24 h after admission and before the observation of a positive blood culture on prognosis. (1) The three most frequently detected bacteria in the blood cultures were *Klebsiella pneumoniae*, *Pseudomonas aeruginosa*, and *Acinetobacter baumannii*, and the amount of *K. pneumoniae* detected increased gradually. (2) Compared with the group of patients who were negative for bloodstream infection , the positive bloodstream infection group had a larger total body burn surface area (TBSA) (*t* = − 5.097, *P* = 0.000) and third-degree burn area (*t* = − 5.133, *P* = 0.000), a significantly longer length of hospital stay (*t* = 3.003, *P* = 0.003) and the length of ICU stay (*t* = 4.258, *P* = 0.000), and a significantly higher rate of in-hospital mortality (*χ*^2^ = 8.485, *P* = 0.004). When *K. pneumoniae* was detected, the length of hospital stay (*t* = 2.148, *P* = 0.035) and the length of ICU stay (*t* = 2.880, *P* = 0.005) were significantly prolonged. (3) The two antibiotics that were most frequently used in patients with acute burns within 24 h after admission were lincomycin (90 cases, 29.32%) and carbapenems (79 cases, 25.73%). Comparing the clinical characteristics of the lincomycin group and the carbapenem group, the TBSA (*t* = − 3.34, *P* = 0.001) and the third-degree burn area (*t* = − 6.08, *P* = 0.000) of the patients in the carbapenem group were larger, and the length of hospital stay (*t* = − 2.136, *P* = 0.035) and length of ICU stay (*t* = − 5.18, *P* = 0.000) were longer, but the difference in in-hospital mortality was not statistically significant (χ^2^ = 1.983, *P* = 0.159). (4) Comparing the group with appropriate initial antibiotic use within 24 h of admission to the inappropriate use group, the TBSA (*t* = − 0.605, *P* = 0.547), the third-degree burn area (*t* = 0.348, *P* = 0.729), the length of hospital stay (*t* = − 0.767, *P* = 0.445), the length of ICU stay (*t* = − 0.220, *P* = 0.827) and in-hospital mortality (*χ*^*2*^ = 1.271, *P* = 0.260) were not significantly different. (5) Comparing the group with appropriate antibiotic use before a positive blood culture was observed to the group with inappropriate antibiotic use, the TBSA (*t* = − 0.418, *P* = 0.677), the third-degree burn area (*t* = 0.266, *P* = 0.791), the length of hospital stay, the length of ICU stay (*t* = 0.995, *P* = 0.322) and in-hospital mortality (χ^2^ = 1.274, *P* = 0.259) were not significantly different. We found that patients with a positive blood culture had a larger burn area and a worse prognosis; that the greater the amount of *K. pneumoniae* in the bloodstream of burn patients was, the longer the hospital and ICU stays were; that whether appropriate antibiotics were administered to acute critical burn patients 24 h after admission had no effect on the prognosis; and that whether appropriate antibiotics were administered before a positive blood culture was observed had no effect on prognosis.

## Introduction

For critically ill patients, infections, especially multidrug-resistant bacterial infections, are often associated with higher mortality, longer hospital stays, and higher medical costs because there are fewer options for antibiotic treatment^1,2^. However, it has been reported that many antibiotics used for anti-infection treatment are inappropriate^3^, which can easily lead to bacterial resistance^4^, poor prognosis^5^, and high mortality^6^. Among all types of infections, bloodstream infections have the highest mortality rate^7^, which makes anti-infection treatment difficult. For burn patients, due to the loss of the skin barrier and the reduction in their immune defence ability^8,9^, large amounts of antibiotics and invasive operations are used during the treatment process^10^, and patients have long hospital stays^11,12^. Infection, especially multidrug-resistant bacteria infection, is the main cause of death in patients with severe burns^13,14^. However, there are few studies on the bacterial epidemiology of blood cultures, the use of antibiotics at admission within 24 h after injury and before positive blood cultures are observed during the treatment of critically ill burn patients or on the effects of these factors on patients’ prognosis. Therefore, we retrospectively studied the clinical data of burn patients with a burn area of more than 50% of the body in our burn ward from 2011 to 2019; in particular, we considered the blood culture results and the use of antibiotics within 24 h after admission and before a positive blood culture was observed and analysed their effects on prognosis. This study provides a basis for the more rational use of antibiotics and the alleviation of bacterial resistance while improving prognosis.

## Objects and methods

This study was a retrospective study. Burn patients who were admitted to the Burn Department of Ruijin Hospital within 24 h after the burn between January 1, 2010, and December 31, 2019, were enrolled in the study. The patients were older than 18 years of age and had a total body burn surface area (TBSA) greater than 50%. The patients’ clinical data were collected from electronic medical records. The exclusion criteria were as follows: (1) Patients who had long-term use of steroids or immunosuppressive agents before their injury; (2) Patients with a history of immunosuppressive diseases.

Observation indicators: General clinical data, including the basic information of the patients enrolled in the study, such as age, sex, burn area and depth, and whether inhalation injury occurred. Prognostic indicators included length of hospital stay, length of intensive care unit (ICU) stay, and in-hospital mortality. The bacterial epidemiological characteristics of the blood culture and the use of antibiotics at the time of admission and before a positive blood culture was observed were collected. Because several kinds of antibiotics were used, the dosage was not listed in detail, and our antibiotics were used according to the recommended dosage in the instructions.

### Definition

Positive blood culture: The positive blood culture results were obtained for blood collected from two or more different sites at the same time. In this study, positive blood culture results were obtained from the patient’s first blood culture results after admission.

Bloodstream infection: Bloodstream infection was defined as a patient with a positive blood culture and corresponding clinical manifestations of systemic infection, such as fever and respiratory rate exceeding 20 breaths/min^15^.

Use of antibiotics at admission: The administration of antibiotics within 24 h after admission.

Initial antibiotic therapy before a positive blood culture was observed: Antibiotics were administered for more than 48 h before positive culture results were obtained^15^.

Appropriate antibiotic therapy: According to the Clinical and Laboratory Standards Institute (CLSI) guidelines^16^ at the time of infection, when the isolated pathogen is sensitive to the initial antibiotic used in vitro, the initial antibiotic therapy is considered appropriate^15,17^.

Data collection and analysis: In this study, the data were statistically processed using IBM SPSS Statistic 21.0 software (IBM Corporation, Armonk, NY, USA). Measurement data that conformed to a normal distribution are described as xˉ ± s. The annual variation in the bacterial composition ratio in the blood cultures of burn patients was analysed by Curve Estimation. Burn area, third-degree burn area, and length of hospital and ICU stays were compared using the independent sample t-test, and the χ^2^ test was used to compare in-hospital mortality. All statistical tests were 2-tailed and were considered statistically significant if *p* < 0.05.

As part of the Clinical Research Plan of SHDC (No. SHDC2020CR1028B), this project was approved by Ruijin Hospital Ethics Committee, Shanghai Jiaotong University School of Medicine. Data privacy was protected according to ethical guidelines. The grant number was (2020) Clinical Ethics Review No. (221).

### Ethical approval and consent to participate

It was approved by Ruijin Hospital Ethics Committees, Shanghai Jiaotong University School of Medicine. Data privacy was protected according to ethical guidelines. The grant number was (2020) Clinical Ethics Review No. (221). The need for informed consent was waived by Ruijin Hospital Ethics Committees, Shanghai Jiaotong University School of Medicine since this is a retrospective study.

## Results

### General information

A total of 307 patients with an average age of 44.30 ± 13.75 years old were enrolled, including 56 females (18.24%) and 251 males (81.75%). The TBSA was 37.99 ± 26.61%, 188 patients (61.24%) had inhalation injury, 148 patients (48.21%) received mechanical ventilation, the length of hospital stay was 50.36 ± 35.46 days, and the length of ICU stay was 40.32 ± 30.32 days.

### Bacteria in blood culture

Eighty-six patients (28.01%) had positive blood cultures, of whom 66 (76.74%) had one type of bacteria detected in blood cultures, 15 (17.44%) had two types of bacteria detected in blood cultures, and 5 (5.81%) had three types of bacteria detected in the blood cultures. A total of 110 strains were detected, and the three most frequently detected strains were *Klebsiella pneumoniae*, *Pseudomonas aeruginosa*, and *Acinetobacter baumannii* (Table 1). The annual variation in the bacterial composition ratio in the blood cultures of burn patients was analysed, and inverse curve fitting was performed (Table 2). The proportion of *K. pneumoniae* (*P* = 0.038, *R2* = 0.437) showed signs of gradual increase, while the proportions of *P. aeruginosa* (*P* = 0.012, *R2* = 0.570) and *S. aureus* (*P* = 0.009, *R2* = 0.591) gradually decreased.

**Table 1:** Composition ratio of bacteria detected in blood cultures.

Bacterial strain	Number	Proportion (%)
*Klebsiella pneumoniae*	39	35.45
*Pseudomonas aeruginosa*	29	26.36
*Acinetobacter baumannii*	15	13.64
*Enterococcus faecalis*	10	9.09
*Staphylococcus epidermidis*	9	8.18
*Staphylococcus aureus*	4	3.64
*Proteus mirabilis*	2	1.82
*Enterococcus faecium*	1	0.91
*Enterobacter cloacae*	1	0.91
Total	110	100

**Table 2:** Annual changes in the bacterial composition ratios detected in the blood cultures of burn patients.

Year	2010		2011		2012		2013		2014			
	Number of strains	Percentage (%)	Number of strains	Percentage (%)	Number of strains	Percentage (%)	Number of strains	Percentage (%)	Number of strains	Percentage (%)		
*Klebsiella pneumoniae*	0	0.00	3	37.50	2	13.33	3	60.00	3	25.00		
*Pseudomonas aeruginosa*	4	80.00	2	25.00	6	40.00	0	0.00	5	41.67		
*Acinetobacter baumannii*	0	0.00	2	25.00	3	20.00	0	0.00	2	16.67		
*Staphylococcus epidermidis*	0	0.00	0	0.00	0	0.00	1	20.00	1	8.33		
*Staphylococcus aureus*	1	20.00	0	0.00	2	13.33	0	0.00	0	0.00		
*Proteus mirabilis*	0	0.00	1	12.50	0	0.00	0	0.00	0	0.00		
*Enterobacter cloacae*	0	0.00	0	0.00	0	0.00	0	0.00	0	0.00		
*Enterococcus faecalis*	0	0.00	0	0.00	2	13.33	1	20.00	1	8.33		
*Enterococcus faecium*	0	0.00	0	0.00	0	0.00	0	0.00	0	0.00		

Year	2015		2016		2017		2018		2019		Inverse model	
	Number of strains	Percentage (%)	Number of strains	Percentage (%)	Number of strains	Percentage (%)	Number of strains	Percentage (%)	Number of strains	Percentage (%)	*P* value	*R*^*2*^
*Klebsiella pneumoniae*	2	28.57	7	41.18	6	66.67	7	38.89	6	42.85	0.04	0.44
*Pseudomonas aeruginosa*	3	42.86	2	11.76	2	22.22	3	16.67	2	14.29	0.01	0.57
*Acinetobacter baumannii*	0	0.00	2	11.76	1	11.11	2	11.10	3	21.43	0.52	0.05
*Staphylococcus epidermidis*	2	28.57	2	11.76	0	0.00	1	5.56	2	14.29	0.21	0.19
*Staphylococcus aureus*	0	0.00	0	0.00	0	0.00	1	5.56	0	0	0.01	0.59
*Proteus mirabilis*	0	0.00	0	0.00	0	0.00	1	5.56	0	0	0.67	0.02
*Enterobacter cloacae*	0	0.00	0	0.00	0	0.00	1	5.56	0	0	0.52	0.05
*Enterococcus faecalis*	0	0.00	3	17.65	0	0.00	2	11.10	1	7.14	0.29	0.14
*Enterococcus faecium*	0	0.00	1	5.89	0	0.00	0	0.00	0	0	0.60	0.04

### The relationship between bloodstream infection and prognosis

This study found that compared with the burn patients in the negative blood culture group, the burn patients in the positive blood culture group had a larger TBSA (*t* = − 5.097, *P* = 0.000) and third-degree burn area (*t* = − 5.133, *P* = 0.000), a significantly longer length of hospital stay (*t* = 3.003, *P* = 0.003) and length of ICU stay (*t* = 4.258, *P* = 0.000), and a significantly higher in-hospital mortality rate (χ^2^ = 8.485, *P* = 0.004) (Table 3). Further analysis of the bacteria detected in the positive blood culture group showed that the length of hospital stay (*t* = 2.148, *P* = 0.035) and the length of ICU stay (*t* = 2.880, *P* = 0.005) were significant longer in patients whose blood cultures were positive for *K. pneumoniae* than in those whose blood cultures were negative for *K. pneumoniae*. There was no significant difference between patients with positive and negative results for other bacteria (*P* > 0.05, Table 4). A comparison of the in-hospital mortality of the patients with positive blood culture results for the three most frequently detected bacteria with that of the negative patients (Table 5) showed that whether the detected bacteria were *K. pneumoniae* (χ^2^ = 0.518, *P* = 0.472), *P. aeruginosa* (χ^2^ = 1.961, *P* = 0.161) or *A. baumannii* (χ^2^ = 0.239, *P* = 0.625) had no effect on in-hospital mortality.

**Table 3:** Relationship between blood culture results and the prognosis of burn patients.

Grouping	Number of cases	TBSA (%)	Third-degree burn area (%)	Length of hospital stay	Length of ICU stay	In-hospital mortality	
						Number of deaths	Number of survivors
Positive group	86	77.52 ± 14.26	50.00 ± 24.87	61.11 ± 41.65	52.85 ± 33.85	20 (23.26%)	66 (76.74%)
Negative group	221	68.17 ± 14.50	33.31 ± 28.84	46.17 ± 31.87	35.44 ± 27.39	23 (10.41%)	198 (89.59%)
Statistical value		*t* = − 5.097	*t* = − 5.133	*t* = 3.003	*t* = 4.258	*χ*^2^ = 8.485	
*P* value		0	0	0.003	0	0.004

**Table 4:** The effect of different bacteria in blood culture on the length of hospital and ICU stays of burn patients.

	Positive group	Negative group	*t*	*P*
***Klebsiella pneumoniae***				
Length of hospital stay	71.49 ± 47.66	52.51 ± 34.10	2.148	0.035
Length of ICU stay	63.92 ± 33.96	43.66 ± 31.22	2.880	0.005
***Pseudomonas aeruginosa***				
Length of hospital stay	61.55 ± 37.50	60.89 ± 43.93	0.069	0.945
Length of ICU stay	53.14 ± 38.78	52.70 ± 31.41	0.056	0.955
***Acinetobacter baumannii***				
Length of hospital stay	51.27 ± 30.04	63.20 ± 43.60	−1.008	0.316
Length of ICU stay	46.73 ± 30.98	54.14 ± 34.49	−0.768	0.444
***Staphylococcus epidermidis***				
Length of hospital stay	55.11 ± 16.57	61.82 ± 43.67	−0.445	0.650
Length of ICU stay	49.33 ± 15.42	53.26 ± 35.42	−0.328	0.744
***Staphylococcus aureus***				
Length of hospital stay	67.00 ± 49.50	60.83 ± 41.57	0.288	0.774
Length of ICU stay	36.00 ± 11.63	53.67 ± 34.39	−1.02	0.311
***Proteus mirabilis***				
Length of hospital stay	79.00 ± 26.87	60.69 ± 41.95	0.612	0.542
Length of ICU stay	69.00 ± 41.01	52.46 ± 33.86	0.681	0.498
***Enterobacter cloacae***				
Length of hospital stay	54.00	60.69 ± 41.95	0.640	0.542
Length of ICU stay	54.00	52.84 ± 34.05	0.839	0.498
***Enterococcus faecalis***				
Length of hospital stay	39.00 ± 16.57	61.20 ± 46.89	−1.810	0.074
Length of ICU stay	34.00 ± 17.59	55.33 ± 34.75	−1.902	0.061
***Enterococcus faecium***				
Length of hospital stay	31.00	61.47 ± 41.77	−0.725	0.470
Length of ICU stay	31.00	53.11 ± 33.97	−0.647	0.519

**Table 5:** Effects of the three most frequently detected bacteria in blood culture on in-hospital mortality of burn patients.

Bacterial group	Number of deaths	Number of survivors	*χ*^2^	*P* value
*K. pneumoniae* group	10(25.64%)	29(74.36%)	0.518	0.472
Non-*K. pneumoniae* group	14(19.72%)	57(80.28%)		
*P. aeruginosa group*	9(31.03%)	20(68.97%)	1.961	0.161
Non-*P. aeruginosa* group	15(18.52%)	66(81.48%)		
*A. baumannii* group	4(26.67%)	11(73.33%)	0.239	0.625
Non-*A. baumannii* group	20(21.05%)	75(78.95%)		

### Initial antibiotic therapy at admission

The two most frequently used antibiotics used within 24 h after admission were lincomycin (90 cases, 29.32%) and carbapenems (79 cases, 25.73%) (Table 6). In the positive blood culture group, the two most frequently used antibiotics were carbapenems (30 cases, 34.88%) and lincomycin (19 cases, 22.09%). In the negative blood culture group, the two most frequently used antibiotics were lincomycin (71 cases, 32.13%) and carbapenems (49 cases, 22.17%). We further analysed the clinical characteristics of 169 patients who were initially treated with carbapenem after admission (Table 7). The results showed that compared with the lincomycin group, the patients in the carbapenem group had a larger TBSA (*t* = − 3.34, *P* = 0.001) and third-degree burn area (*t* = − 6.08, *P* = 0.000) and longer hospital (*t* = − 2.136, *P* = 0.035) and ICU stays (*t* = − 5.18, *P* = 0.000), and the differences between the groups were significant. However, the in-hospital mortality rate was 13.33% (12/90) in the lincomycin group and 21.52% (17/79) in the carbapenem group, and there was no significant difference between the two groups (*Χ*^2^ = 1.983, *P* = 0.159).

**Table 6:** Initial antibiotic therapy within 24 h after admission (n = 307).

Type of antibiotic	Positive blood culture N (%)	Negative blood culture (%)	Total (%)
Lincomycin	19 (22.09)	71 (32.13)	90 (29.32)
Carbapenems	30 (34.88)	49 (22.17)	79 (25.73)
Second-generation cephalosporins	13 (15.12)	29 (13.12)	42 (13.68)
Aminoglycoside + first-generation cephalosporins	5 (5.81)	20(9.05)	25 (8.14)
Third-generation cephalosporins	9 (10.47)	22 (9.95)	31 (10.09)
First-generation cephalosporins	4 (4.65)	12 (5.43)	16 (5.21)
Penicillin	2 (2.33)	9 (4.07)	11(3.58)
Aminoglycosides	0	3 (1.36)	3 (0.98)
Glycopeptides	2 (2.33)	2 (0.90)	4 (1.30)
Aminoglycoside + second-generation cephalosporins	1 (1.16)	1(0.45)	2 (0.65)
Penicillin + third-generation cephalosporins + glycopeptides	1 (1.16)	0	1 (0.33)
Glycopeptides + third-generation cephalosporins	0	3 (1.35)	3 (0.98)
Total	86 (100)	221 (100)	307 (100)

**Table 7:** Comparison of clinical characteristics between the lincomycin group and the carbapenem group (n = 169).

Classification	TBSA (%)	Third-degree burn area (%)	Length of hospital stay (days)	Length of ICU stay (days)	In-hospital mortality	
					Number of deaths	Number of survivors
Lincomycin group(n = 90)	69.41 ± 13.68	26.13 ± 25.02	45.82 ± 26.19	27.53 ± 15.27	12(13.33%)	78 (86.67%)
Carbapenem group (n = 79)	76.78 ± 15.04	50.84 ± 27.79	58.08 ± 44.75	49.63 ± 35.16	17(21.52%)	62(78.48%)
Statistical value	*t* = − 3.34	*t* = − 6.08	*t* = − 2.136	*t* = − 5.18	*χ*^2^ = 1.983	
*P* value	0.001	0.000	0.035	0.000	0.159	

### Effect on prognosis of the initial use of appropriate antibiotics within 24 h after admission in patients with positive blood culture

Among the 86 patients with positive blood cultures, 4 (4.65%) received initial treatment with appropriate antibiotics within 24 h after admission, and 82 (95.35%) received inappropriate antibiotics. Further analysis of the relationship between treatment with appropriate antibiotics within 24 h after admission and prognosis (Table 8) showed that there were no significant differences in the TBSA (*t* = − 0.605, *P* = 0.547), third-degree burn area (*t* = 0.348, *P* = 0.729), length of hospital stay (*t* = − 0.767, *P* = 0.445), length of ICU stay (t = − 0.220, *P* = 0.827), and in-hospital mortality (χ2 = 1.271, *P* = 0.260) between patients who received appropriate antibiotics and those who received inappropriate antibiotics.

**Table 8:** The effect on prognosis of initial treatment with appropriate antibiotics within 24 h after admission among patients with positive blood cultures.

Classification	TBSA (%)	Third-degree burn area (%)	Length of hospital stay	Length of ICU stay	In-hospital mortality	
					Number of deaths	Number of survivors
Appropriate antibiotic group (n = 4)	81.75 ± 12.87	45.75 ± 21.42	76.75 ± 38.04	56.50 ± 23.10	0 (0.00%)	4 (100.00%)
Inappropriate antibiotic group (n = 82)	77.32 ± 14.36	50.21 ± 25.12	60.35 ± 41.88	52.67 ± 34.48	20 (24.39%)	62 (75.61%)
Statistical value	*t* = − 0.605	*t* = 0.348	*t* = − 0.767	*t* = − 0.220	*χ*^2^ = 1.271	
*P* value	0.547	0.729	0.445	0.827	0.260	

### The effect of treatment with appropriate antibiotics prior to the observation of a positive blood culture on prognosis

Among the 86 patients with positive blood cultures, 16 (18.60%) received appropriate antibiotics before a positive blood culture was observed, and 70 (81.40%) received inappropriate antibiotics. Further analysis of the relationship between treatment with appropriate antibiotics before a positive blood culture was observed and prognosis (Table 9) showed that there were no significant differences in the TBSA (*t* = − 0.418, *P* = 0.677), third-degree burn area (*t* = 0.266, *P* = 0.791), length of hospital stay (*t* = 0.014, *P* = 0.989), length of ICU stay (*t* = 0.995, *P* = 0.322), and in-hospital mortality (*χ2* = 1.274, P = 0.259) between the appropriate and inappropriate antibiotic groups.

**Table 9:** The effect on prognosis of treatment with appropriate antibiotics before a positive blood culture was observed.

Classification	TBSA (%)	Third-degree burn area (%)	Length of hospital stay	Length of ICU stay	Hospital mortality	
					Number of deaths	Number of survivors
Appropriate antibiotic group (n = 16)	78.88 ± 14.38	48.50 ± 20.04	61.00 ± 34.96	45.25 ± 21.86	2(12.50%)	14 (87.50%)
Inappropriate antibiotic group (n = 70)	77.21 ± 14.31	50.34 ± 25.96	61.14 ± 43.26	54.59 ± 35.93	18(25.71%)	52(74.29%)
Statistical value	*t* = − 0.418	*t* = 0.266	*t* = 0.014	*t* = 0.995	*χ*^2^ = 1.274	
*P* value	0.677	0.791	0.989	0.322	0.259	

## Discussion

Infection, especially bloodstream infection, is the main cause of death in burn patients^18^. With the increasing use of antibiotics, bacterial resistance in burn patients is gradually becoming severe^11^. If bacterial resistance is not controlled, there may be no drugs available in the future to treat drug-resistant bacteria infections^19^. In anti-infective therapy, some antibiotics are used inappropriately^3^, which can lead to an increase in the mortality of critically ill patients infected with drug-resistant bacteria^6^. However, for patients with critical burns, there are few studies on whether treatment with appropriate antibiotics at admission within 24 h after injury and before positive blood cultures are observed affects prognosis. Therefore, we investigated the blood culture results of burn patients with a burn area of more than 50% who were seen in our burn ward from 2011 to 2019 and the use of antibiotics at admission and before positive blood cultures observed and analysed the effect of appropriate antibiotics use on the prognosis. Our findings provide a basis for the more rational use of antibiotics in the future.

This study found that most of the bacteria detected in the bloodstream infections of burn patients were gram-negative, similar to other reports^20^. Due to the increased risk of poor prognosis in patients with blood cultures that re positive for gram-negative bacteria^21^, it is important to pay attention to this phenomenon. Further analysis of bacterial species showed that the top three most abundant bacteria detected were *K. pneumoniae*, *P. aeruginosa*, and *A. baumannii*. Furthermore, the proportions of *K. pneumoniae* (*P* = 0.038, *R*^*2*^ = 0.437) showed signs of progressive increase, which is similar to the findings of other studies^22^. It has been reported that the increase in the detection of *K. pneumoniae* may be related to the use of large amounts of antibiotics^23^. There are fewer effective antibiotics for *K. pneumoniae* infection, especially multidrug-resistant *K. pneumoniae* infection^24^, which can increase the in-hospital mortality of patients^25^. Therefore, we must pay attention to avoiding the unreasonable use of antibiotics for the treatment of *K. pneumoniae*, especially multidrug-resistant strains. More importantly, the rate of *K. pneumoniae* detected overall in the burn ward also showed an increasing trend^10^ similar to the trend observed in blood cultures, suggesting that we need to strengthen wound treatment to reduce the bacterial load of wounds. At the same time, attention should be paid to catheter care and disinfection and isolation measures to prevent wound bacteria from entering the bloodstream.

Combining blood culture results with the TBSA, the third-degree burn area, the length of hospital stay, the length of ICU stay, and the in-hospital mortality, this study found that patients with positive blood cultures had a greater TBSA and third-degree burn area, longer hospital and ICU stays, and higher mortality than patients with negative blood cultures, indicating that patients with positive blood cultures had more severe illness and a worse prognosis. However, it has also been reported that a positive blood culture is not associated with mortality in patients with severe sepsis^26^, possibly because when the patient reaches the late stage of severe sepsis, the functional deterioration of the various organ systems affects prognosis more than the infection factors do. This also indicates that the earlier a positive blood culture is detected, the earlier the intervention takes places, which may be more conducive to the prognosis, and the timely performance of a blood culture can affect patient survival more than the blood culture results themselves. Therefore, we need to pay attention to the timing of blood cultures, perform blood cultures in a timely manner and adjust the antibiotic regimen according to the changes in the disease condition^27^.

Further analysis of the effects of different strains on prognosis showed that patients with blood cultures positive for *K. pneumoniae* had longer hospital and ICU stays than those with blood cultures that were positive for other strains, a finding that may be related to the high virulence of *K. pneumoniae*; furthermore, the initial antibiotic treatment was inappropriate in most cases^28^. Therefore, during the treatment process, we need to pay attention to the treatment of patients with *K. pneumoniae*-positive blood cultures and use appropriate antibiotics. However, the analysis of the effect of different bacterial species on the in-hospital mortality of burn patients showed that compared with other bacteria, the three bacteria most commonly detected in blood culture, *K. pneumoniae* (*χ*^*2*^ = 0.518, *P* = 0.472), *P. aeruginosa* (*χ*^*2*^ = 1.961, *P* = 0.161), and *A. baumannii* (*χ*^*2*^ = 0.239, *P* = 0.625), had no effect on in-hospital mortality. This may be because the patients enrolled in this study were all critically ill burn patients. Unlike patients with other diseases, severe burn patients have poor tolerance of bloodstream infections due to the loss of the skin barrier and decreased immunity^8,9^. No matter what kind of bacterial infection leads to bloodstream infection, it will have a negative impact on the prognosis of patients.

To prevent infection, burn patients within 24 h after burn often receive antibiotics within 24 h after admission to our department. According to the statistics for antibiotics administration at the time of admission to our ward for the positive blood culture and negative blood culture groups, the two most frequently used antibiotics were lincomycin and carbapenems, accounting for nearly 50% of antibiotic treatments. Further analysis of the characteristics of patients in the lincomycin group and the carbapenem group showed that compared with the lincomycin group, the carbapenem group had a larger TBSA and area of third-degree burns. This indicates that the larger the burn area is, the higher the probability of developing a bloodstream infection during the treatment process^29^. Therefore, we prefer to use carbapenem antibiotics to prevent infection at admission. However, when we compared the prognosis of the two groups, we found that although the length of hospital stay and the length of ICU stay in the carbapenem group were longer, there was no difference in mortality between the two groups. This indicates that even though the patients in the carbapenem group had a larger TBSA and more severe disease, the use of prophylactic carbapenems or lincomycin at admission had no effect on mortality. Further analysis of the relationship between the appropriate use of antibiotics and blood culture results and patient prognosis showed that for 4.65% of patients were treated with appropriate antibiotics, which was a very low proportion. There was no significant difference in the TBSA or the third-degree burn area between the two groups of patients. In this case, the appropriateness of the antibiotics also did not affect the length of hospital stay, the length of ICU stay, or the mortality rate. This indicates that for patients with acute burns, whether the antibiotic treatment within 24 h after admission is appropriate for the subsequent bloodstream infections has no effect on prognosis. In fact, no matter whether the prophylactic use of antibiotics is “appropriate” or not, it is actually not appropriate, which does not improve the prognosis, but increases the risk of irrational antibiotic use and bacterial resistance.

Thus, it is worth reflecting on whether it is necessary to administer carbapenems and other antibiotics within 24 h after admission for patients with acute and critical burns. Although the larger the burn area is, the higher the probability of bloodstream infection^29^, and although the literature reports that the early use of antibiotics in patients with sepsis could reduce mortality^30^, the presence of early acute and critical burns does not mean that patients are severely infected, and prophylactic antibiotics, especially carbapenem antibiotics, should not be administered. In addition, the extensive use of carbapenem antibiotics is likely to cause bacterial resistance^31^ and increase the in-hospital mortality of patients^32^. The International Society for Burn Injury (ISBI) also suggested that there is currently no strong evidence that the use of prophylactic antibiotics for acute burn patients at an early stage is beneficial; in contrast, it may lead to the emergence of drug-resistant strains, diarrhoea, *Clostridium difficile* infection, allergic reactions, and liver, kidney, or bone marrow toxicity, and therefore, the prophylactic use of antibiotics is not recommended in the early stage of acute burns^33^. However, ISBI^33^ also acknowledges that there are few strong relevant studies at present, and many burn doctors choose to use antibiotics for prophylaxis for acute burn patient ^34^. Especially in China, many burn doctors are still used to using antibiotics for prophylaxis for acute burn patient ^35^. But, our study supports this recommendation of ISBI. Consequently, we need to change the antibiotic use strategy for patients with acute burns at admission to avoid the use of carbapenem or even avoid antibiotics use altogether. Of course, after admission, we also need to closely observe changes in patients’ microbial detection and inflammatory indicators and adjust the antibiotic treatment strategy in a timely manner during the treatment process. However, since this was a single-centre study, it is still debatable whether this result can be extended to other hospitals. Ward cleanliness, bacterial epidemiology, hand hygiene and other nosocomial infection control measures are different at each hospital and may affect the decision to administer antibiotics in the early stage of acute burns. However, at our hospital, Ruijin Hospital, which is a top hospital in Shanghai, China, it is not recommended to use prophylactic antibiotics for patients with acute burns in the early stage. And we hope our study will help burn doctors in China change their strategies for the application of antibiotics for prophylaxis for acute burn patient. We hope prophylactic antibiotics are stopped our resistant organisms will be reduced.

It has been reported that for the anti-infection treatment of drug-resistant bacterial strains, the initial use of many antibiotics is not appropriate, but the use of appropriate antibiotics can reduce the mortality of patients with infection^28^. Therefore, for patients with positive blood cultures, the use of appropriate initial antibiotics is very important. Surprisingly, after analysing the use of antibiotics before a positive blood culture was observed, we found that when there was no significant difference in the TBSA and the third-degree burn are between the two groups of patients, whether the antibiotics that were used were appropriate had no effect on the length of hospital stay, the length of ICU stay, or in-hospital mortality. However, it has previously been reported that whether or not the initial antibiotic used for in patients with infection is appropriate is not associated with mortality^36,37^ and that the use of inappropriate initial antibiotics in infected patients leads to an increase in mortality^25,38^. Such contradictory results may be explained as follows: 1. In addition to the selection of appropriate antibiotics, whether anti-infective treatment is effective is also related to the specific bacterial resistance and the method of antibiotic administration^25^. If the bacteria are multidrug-resistant strains, anti-infection treatment can easily fail. In addition to the choice of antibiotics, the dose, course of treatment, and drug concentration at the site of action also have an important impact on the prognosis. 2. Our study examined the appropriateness of antibiotic treatment before a positive blood culture was observed, and the timing of blood cultures often depends on the clinical judgement of the physician. Delays in performing blood cultures will lead to the delay of anti-infection treatment and an increase patient mortality^29^. Therefore, it is possible that a late blood culture may lead to delays in treatment and aggravation of the disease, resulting in a poor prognosis even when the initial antibiotics are appropriate. 3. More importantly, burn infections are different from internal medicine infections. It has been reported^39^ that the appropriate use of antibiotics before surgery in patients with acute appendicitis does not affect surgical site infection or the length of hospital stay. For surgical infections, surgery may be more important than antibiotic treatment. Similarly, burn infections have their own characteristics. Due to the poor distribution of antibiotics in burn wounds, the use of antibiotics alone cannot prevent and treat burn wound infections and will promote the emergence of multidrug-resistant bacteria^40^. Moreover, regardless of whether a wound infection or bloodstream infection is present, the administration of antibiotics is only a single important part of the anti-infection treatment strategy for burn patients. Measures such as wound care, surgical management of the burn wound, and nosocomial infection control also play an important role in the prognosis of burn patients^33^.

This study has some limitations. (1) This study was a retrospective study that relied on electronic medical records. Some important information could not be accurately obtained, and some uncontrolled factors might affect the results. (2) This was a single-centre study, and the results may not be extendable to other centres and settings. (3) The choice of antibiotics is only a factor that affects the prognosis of burn patients with bloodstream infection. Therefore, it may lead to the result that whether antibiotics are appropriate before positive blood culture observed has no effect on the prognosis. In the future, it is necessary to further study the effect of antibiotics on the prognosis of burn patients. (4) We did not analyse the mortality in the first few days of admission, but the in-hospital mortality. Therefore, it is not known whether the use of antibiotics prophylaxis within 24 h after admission has an impact on the early mortality of admission.

In summary, our study confirmed that patients with positive blood cultures had a larger burn area and a worse prognosis. In recent years, Klebsiella pneumoniae has been detected more and more in blood stream infection of burn patients, with longer hospital stay and ICU days. Although a larger burn area increases the possibility of developing a bloodstream infection during the treatment process, antibiotics—especially carbapenem antibiotics—are not recommended for prophylaxis for patients with acute and critical burns after admission within 24 h after burn. For critically ill burn patients, the administration of appropriate antibiotics before a positive blood culture is observed has no effect on the prognosis. In the future, we will design further prospective studies to understand the effects of the type of antibiotics, method of administration, and tissue concentration on the prognosis of critically ill burn patients with blood infections to provide a reference for the more reasonable use of antibiotics, the improvement of patient prognosis, and the avoidance of bacterial resistance.

## Data Availability

All data requests should be submitted to Dou Yi (douyi815@hotmail.com) for consideration.
